# Listener characteristics modulate the semantic processing of native vs. foreign-accented speech

**DOI:** 10.1371/journal.pone.0207452

**Published:** 2018-12-05

**Authors:** Rebecca Holt, Carmen Kung, Katherine Demuth

**Affiliations:** 1 Department of Linguistics, Macquarie University, Sydney, New South Wales, Australia; 2 ARC Centre of Excellence in Cognition and its Disorders, Macquarie University, Sydney, New South Wales, Australia; Edge Hill University, UNITED KINGDOM

## Abstract

Foreign accents have been shown to have considerable impact on how language is processed [1]. However, the impact of a foreign accent on *semantic* processing is not well understood. Conflicting results have been reported by previous event-related potential (ERP) studies investigating the impact of foreign-accentedness on the N400 effect elicited by semantic violations. Furthermore, these studies have only examined a subset of the four characteristics of the N400 (i.e. onset latency, latency, amplitude, and scalp distribution), and have been conducted in linguistic environments where foreign-accented speech is relatively uncommon. The current study therefore compared the N400 effect elicited by semantic violations in native Australian English vs. Mandarin-accented English, in a context where foreign-accented speech is common. Factors which may be responsible for individual variability in N400 amplitude were also investigated. The results showed no differences between the N400s elicited by native and foreign-accented speech in any of the four aforementioned characteristics. However, the analysis of individual variability revealed an effect of familiarity with foreign-accented speech on the amplitude of N400 effects for semantic violations. An effect of working memory capacity on N400 amplitude was also found. These findings highlight the relevance of the ambient linguistic environment for studies of speech processing, and demonstrate the interacting influences of both speaker- and listener-related factors on semantic processing.

## Introduction

### Semantic processing of foreign-accented speech

In our globalised world it is common for language users to encounter speakers of their language who have a foreign accent. This is particularly the case with languages such as English, which are commonly used as a lingua franca among speakers of diverse linguistic backgrounds. Worldwide, 64% of English speakers are non-native speakers of English [2], and thus have a high likelihood of speaking with a foreign accent [3]. Even in predominantly English-speaking countries, the percentage of immigrant, and therefore potentially foreign-accented, speakers can be substantial. In Sydney, Australia, for example, 42% of the population are born outside Australia [4].

An understanding of how foreign-accented speech is processed is therefore essential for an ecologically valid picture of language processing. The impact of a foreign accent on some aspects of speech processing has been examined in detail (particularly phonological processing), while other aspects remain little studied. One such area is semantic processing, which involves the online process of retrieving and integrating the meaning of lexical items.

A small number of studies have used event-related potentials (ERPs) and the semantic-violation paradigm to explore the semantic processing of foreign-accented speech [1,5,6]. ERPs enable investigation of listeners’ brain responses to language input with excellent temporal precision via the measurement of scalp-recorded electrical activity. The resulting ERP component can be described in relation to the following characteristics: polarity, amplitude, latency, onset latency and topographical distribution. ERP components can be correlated to specific cognitive behaviours associated with the processing of the stimulus [7]. In ERP studies of semantic processing, a semantic-violation paradigm is typically employed in which participants read or listen to words that are either semantically congruent or incongruent with the preceding sentential/discourse context while their brain activity is recorded (e.g. ‘cupcake’ in *The lady bites the* ***cupcake*** vs. ‘suitcase’ in The lady bites the *suitcase). Generally, semantically incongruent sentences elicit a larger negativity than semantically congruent sentences, peaking around 400 ms post-stimulus-onset and with a centro-parietal maximum ([8], see [9] for a review). This negativity is referred to as the N400 component and the difference in N400 amplitude between two conditions is termed an N400 effect. This effect is generally interpreted as being an index of semantic-memory retrieval or semantic integration difficulty [9,10].

There appears to be a consensus that the N400 effects elicited by semantic violations in native and foreign-accented speech differ. The nature of the difference has varied across studies but has primarily involved the amplitude and topography of the N400. Hanuliková and colleagues [1] found that the N400 had a broader topographical distribution in response to foreign-accented than native Dutch, but did not differ in amplitude. In partial contrast, Romero-Rivas, Martin and Costa [5] found that the N400 effect for foreign-accented Spanish was both more broadly distributed and of greater amplitude than that observed for native Spanish. Finally, Grey and Van Hell [6] found that the N400 effect for foreign-accented speech had a later onset latency than that for native English, but were unable to directly compare the two N400s in amplitude and topography as they occurred in different time windows. (Note that due to this timing difference the N400 for foreign-accented speech could alternatively be interpreted as a functionally distinct late negativity).

Aside from these studies of N400 effects elicited by semantic violations, there has also been some examination of N400 effects elicited by foreign-accented sentences varying in cloze probability and in semantically congruent sentences varying in accentedness. Regarding cloze probability, similar N400 effects were found when comparing foreign-accented sentences with the semantic best completion to sentences with both semantically related and unrelated completions [11]. This differed from the pattern for native speech, which saw a gradient effect of semantic relatedness on N400 amplitude. Regarding sentence accentedness, an N400 effect was also found when comparing semantically congruent sentences produced in a native accent to semantically congruent sentences produced with a foreign accent [12]. However, as these studies examined N400 effects elicited by phenomena other than semantic violations, they are not directly comparable to the studies outlined above, or the current study.

Previous results thus consistently demonstrate a difference in the N400 effects elicited by semantic violations in native and foreign-accented speech, but *how* and *where* this is realised across studies has been inconsistent. Furthermore, no studies have set out to investigate component latency or onset latency differences. *Component latency* here refers to the duration of the ERP effect from onset to offset, while *onset latency* refers to the time-point (measured from the time-locking point) at which the difference between conditions begins to be significant [7]. The above studies all employed pre-defined time-windows or time-windows based on visual inspection of the waveforms when analysing ERP data, making it impossible to observe temporal differences between conditions. While a difference in onset latency was observed between N400s in [6], this was essentially a side-effect of employing consecutive N400 and P600 time windows. If an analysis method were used that enabled statistical comparison of component latency and onset latency more deliberately, one might expect to observe more fine-grained differences between native and foreign-accented speech on these temporal dimensions. More specifically, given that lexical retrieval seems to occur more slowly in foreign-accented than native speech [13], one might expect N400s for foreign-accented speech to exhibit a longer onset latency than their native-speech counterparts, as was found on a gross scale in [6].

Another limitation shared by these previous studies is that all were conducted in cities or states with relatively small immigrant populations. Grey and van Hell [6] was conducted in central Pennsylvania, USA, a state in which 94% of the population are American-born [14]; Hanuliková et al. [1] was conducted in Nijmegen, The Netherlands, where 75% of the population are at least second-generation Dutch [15]; and Romero-Rivas et al.[5] was conducted in Barcelona, where 86% of the population are born in Spain [16]. Note that one could argue that, because Catalan and Spanish are both official languages of Barcelona, it may be assumed that residents of Barcelona are regularly exposed to Catalan-accented Spanish. However, we do not consider Catalan-accented speakers of Spanish to be ‘foreign-accented’ for the purposes of our study, due to Catalan’s official-language status (i.e., it is not ‘foreign’). Furthermore, in Romero-Rivas et al.’s [5] study, Catalan-accented Spanish was not included as one of the foreign accents under consideration. It is therefore possible that, in the previous studies, unfamiliarity with foreign-accented speech may have affected participants’ neural responses, and that listeners with more extensive experience with foreign-accented speech may show a different pattern of results.

The first aim of the current study was therefore to compare the ERP effects elicited by semantic violations in native and foreign-accented speech (a) using a method that would allow us to deliberately examine differences in component latency and onset latency, as well as amplitude and topography, (b) in a context where participants were likely to be more familiar with foreign-accented speech. Sydney, Australia, was an ideal location for this, as only 58% of Sydney’s population is born in Australia, and 40% of the population is bilingual [4], suggesting that foreign-accented speech is widespread. We hypothesised that, as in previous studies, semantic violations produced by both speakers (native and foreign-accented) would trigger an N400 effect, but that these may show different characteristics. In particular, we expected a possible difference in the N400 effect amplitude between native and foreign-accented speech, and a potentially broader distribution and longer onset latency for N400s in response to foreign-accented speech.

### Individual variability in the N400

Most previous studies investigating the neural correlates of semantic processing in foreign-accented speech have not considered potential sources of individual variability among participants. Yet, the N400 is subject to extensive individual variability [17], which can be attributed to specific characteristics of the listener. For example, the characteristics of N400s observed in response to semantic violations have been shown to covary with participant age [17] and working memory capacity [18,19]. Also, the amplitude of N400s observed in response to speaker incongruities (utterances in which the semantic content of the sentence is plausible in itself but rendered implausible by the identity of the speaker, such as the sentence ‘*I think I might be pregnant’* uttered by a male voice) has been shown to vary as a function of the listener’s empathy level [20].

However, much individual variability in the N400 remains unaccounted for [17], and no study has yet described any sources of individual variability in the neural correlates of processing foreign-accented speech. This is despite behavioural evidence that skill in processing foreign-accented speech is indeed highly variable, and that this variability can, to some extent, be accounted for by participant characteristics [21,22]. While Grey and van Hell [6] attempted to account for individual variability in the N400 effect elicited by foreign-accented speech by measuring language and accent attitudes, cognitive inhibition, working memory, verbal fluency, English proficiency and IQ, no correlations between these factors and N400 amplitude were found to be significant. Therefore, the second aim of the current study, was to investigate other potential sources of individual variability in the amplitude of neural responses to semantic violations in both native and foreign-accented speech. The same factors were considered across both speaker conditions to allow for comparison. These included listeners’ age, gender, working memory capacity, empathy level, how accented they considered the speech to be, and their familiarity with the foreign accent in question. By examining these individual variability factors, the current study investigated not only how speaker-related characteristics, such as a foreign accent, immediately affect semantic processing, but also how these may immediately interact with listener-related characteristics. The potential relevance of both listener- and speaker-related characteristics has implications for neurocognitive models of sentence processing [23–27]. Integration of these characteristics will highlight the immediate use of non-linguistic information during linguistic processing, which is strongly in favour of more interactive models [24–27].

We predicted that working memory capacity would influence N400 amplitude in both native and foreign-accented speech, in line with previous findings for native speech [18,19]. While no significant relationship was found between working memory capacity and N400 amplitude for foreign-accented speech in [6], we still considered it possible that such an effect might be present in our study due to existing behavioural evidence that increased working memory capacity is associated with increased skill in processing foreign-accented speech [22]. In fact, given that processing foreign-accented speech is associated with increased working memory load [28], we expected that the influence of working-memory capacity might in fact be greater when processing the foreign-accented speech than when processing native speech. This relationship between N400 amplitude and working memory capacity is thought to occur due to the ability of listeners to take sentential context into account [19]. As semantic processing necessarily requires integration into sentential context, listeners’ ability to access sentential context may affect the degree of semantic integration difficulty and thus modulate N400 amplitude.

We also predicted that participant empathy scores might influence N400 amplitude, as more empathic listeners may be more lenient towards semantic violations made by non-native speakers, thus displaying a reduced N400 amplitude (cf. [1,29] regarding morphosyntactic violations). Regarding perceived accentedness, stronger accentedness tends to be associated with lower perceived language competence [30]. For this reason, we hypothesised that participants who perceived the foreign-accented speech to be more strongly accented might be more lenient towards the foreign-accented speaker’s errors, and thus show smaller N400 amplitudes in response to the foreign-accented speaker. Finally, participants’ familiarity with the foreign accent was expected to affect the amplitude of the N400 observed for foreign-accented speech, but not native speech. We hypothesised that participants who were more familiar with the accent (determined as those who were able to correctly identify the accent of the speaker, cf. [6]) might also expect semantic errors from the foreign-accented speaker and thus also show a smaller N400 amplitude (cf. [1,29] regarding morphosyntactic violations and [30] regarding phonological mapping).

## Methods

### Participants

Thirty monolingual native speakers of Australian English (13M, 17F) aged 18 to 33 years (Mean = 22 years, SD = 5.3 years) participated in the study. All lived in the greater Sydney area and had parents who were also native speakers of Australian English. The language background of the participants’ parents was controlled in order to avoid effects of exposure to foreign-accented speech (including other dialects of English) during language acquisition, which has been shown to influence speech perception later in life [31]. Participants reported no hearing, language, neurological or cognitive impairments. All were right-handed according to the Edinburgh Handedness Inventory [32]. Data from a further nine participants were excluded from analysis due to technical error (n = 4), having at least one parent who was not a native speaker of Australian English (n = 3), or having a diagnosis of depression or autism spectrum disorder (n = 2). All participants received either course credit or financial reimbursement. The Macquarie University Human Research Ethics Committee approved the experimental methods used and written informed consent was obtained from all participants before testing.

### Materials

#### Preparation of stimuli

Twenty-five pairs of sentences of the form “The [noun] [verb] the [noun]” were created, each pair containing a semantically congruent sentence and a corresponding semantically incongruent sentence (these pairs were termed the ‘semantic sentences’; see S1 Table for the full set of sentences). All verbs were CVC monosyllables and were in the present tense, while all nouns were disyllabic and had initial stress to create a consistent prosodic pattern across sentences. The sentences did not contain any consonant clusters except those created by the addition of the third person singular *-s* where necessary. This was to ensure that the target words were maximally intelligible when produced by the foreign-accented speaker (a native speaker of Mandarin): Consonant clusters, particularly in word-final (coda) position, are difficult to produce for speakers of Mandarin-accented English [33], however it was ensured that any third person singular -*s* occurring in a cluster was indeed produced in the stimuli. All content words in the sentences had a log frequency greater than 3.0 in the SUBTLEX-UK database [34]. All subject nouns were animate (the canonical animacy class for subjects) and consistent thematic roles were maintained across sentences (all subjects were agents, all objects were patients).

Each semantically congruent sentence was recorded by two female speakers. The native speaker was a 24-year-old monolingual speaker of Australian English. The foreign-accented speaker was a 20-year-old native speaker of Mandarin from Beijing who had been living in Australia for two years. This speaker was selected from among three candidates as being the one with the most canonical yet intelligible Mandarin accent. Sentences were elicited from the native speaker using a question and answer method to maintain consistent prosody and to ensure that the final word of each sentence was focused. As all semantic violations occurred sentence-finally, this use of focus served to maximise the salience of the violations. The question-and-answer elicitation method was too challenging for the foreign-accented speaker, so the foreign-accented sentences were elicited by having her imitate a native Australian English speaker (the first author). The two speakers differed in speech rate: Sentences were significantly shorter in duration when produced by the native speaker (Mean = 1.89 s, SD = 0.17 s) than the foreign-accented speaker (Mean = 2.07 s, SD = 0.17 s) according to a paired samples t-test (*t*(49) = 9.24, *p* < .001). However, this was unsurprising as native speakers of Mandarin have previously been found to have a slower speech rate when speaking English than native English speakers [35].

**Fig 1:**
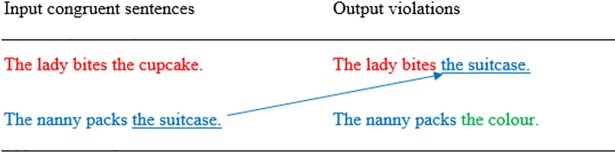
Diagram showing splicing locations for sentences containing semantic violations.

**Fig 2:**
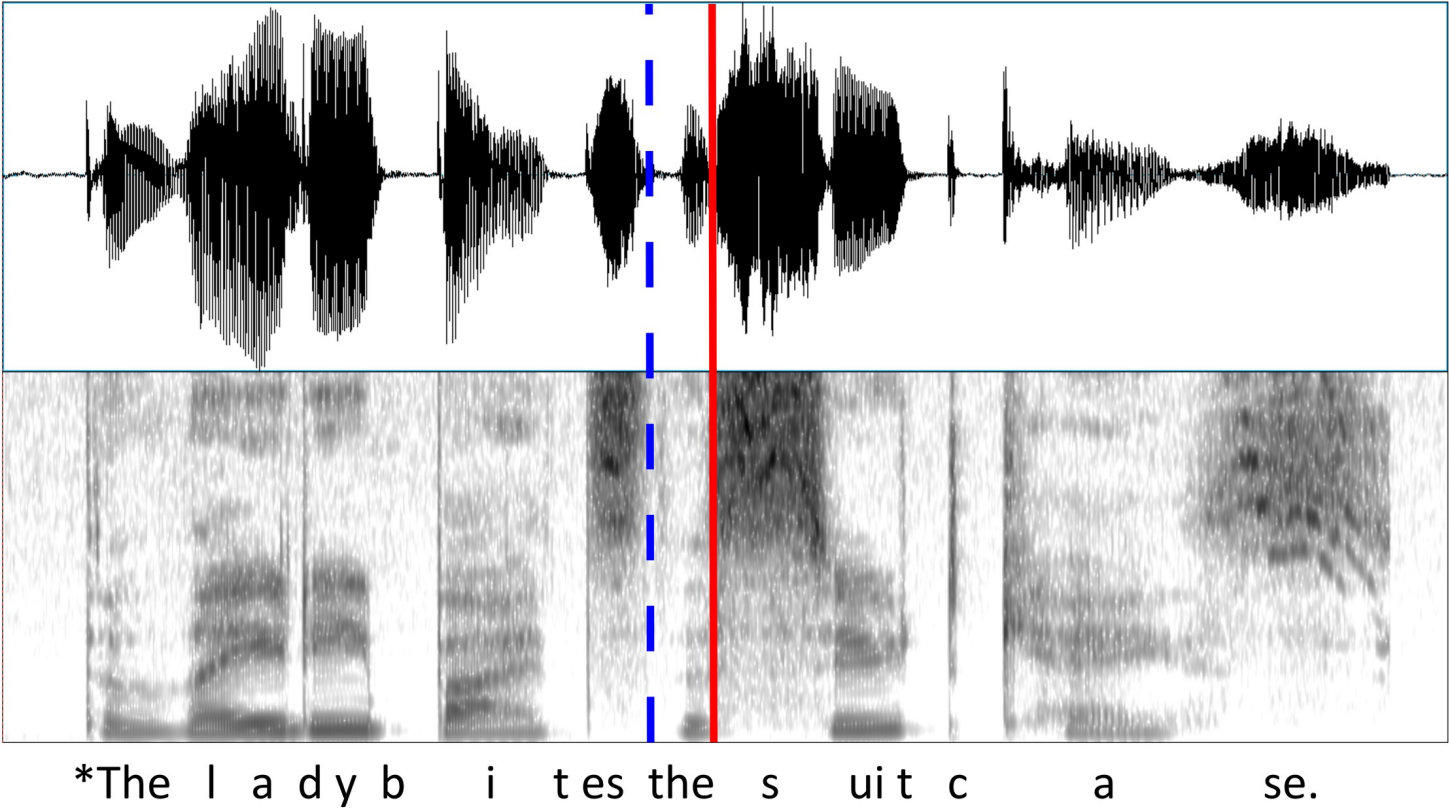
Waveform and spectrogram of sample stimulus sentence “The lady bites the suitcase”. The splicing point is shown as a dotted blue line and the time-locking point of the ERP is shown as a solid red line.

Sentences with semantic violations were created by splicing the semantically congruent sentences at the offset of the verb and appending the incongruent object from another semantically congruent sentence (see Figs 1 and 2). Splicing was conducted using Praat software (Version: 5.4.08) [36] and was used to prevent the appearance of phonetic cues to semantic incongruence occurring before the overt violation [37,38].

In addition to the 100 stimulus sentences (i.e. 25 sentence pairs, with each sentence produced by both speakers), a further 200 sentences used for a different study were included in the EEG task (termed the ‘morphosyntactic sentences’; reported in [29]). These consisted of 100 sentences containing subject-verb agreement violations and 100 corresponding grammatical sentences, and contained the same phonological constraints as the sentences used in the present study (i.e. no consonant clusters except those involving the third person singular *-s*). One hundred filler sentences with no syntactic or phonological constraints were also included. By minimising the phonological constraints placed on the fillers, it was intended that the foreign-accentedness of the Mandarin-accented speaker would be highlighted, thus reinforcing the difference between the two speakers. All of these filler sentences were six or seven syllables in length, and thus similar in length and complexity to the target sentences. Four hundred sentences in total were therefore included in the EEG task (100 semantic sentences, 200 morphosyntactic sentences, and 100 fillers), half produced by each speaker. See Table 1 for a summary of the experimental design.

**Table 1:** Experimental design with example sentences. Target word is in bold.

Conditions		Examples
Speaker	Semantic Congruity	
Native speaker	Congruent	The lady bites the **cupcake**.
	Incongruent	*The lady bites the **suitcase**.
Foreign-accented speaker	Congruent	The lady bites the **cupcake**.
	Incongruent	*The lady bites the **suitcase**.

#### Validation of stimuli

A listening task was conducted to ensure the stimuli were suitable for the EEG task. Ten female native speakers of Australian English aged 19 to 23 years (Mean = 20 years, SD = 1.4 years), all with Australian English-speaking parents, participated for course credit. They reported no hearing, language, neurological or cognitive impairments. First, participants rated all semantic sentences (both congruent and incongruent) produced by the native speaker on a seven-point scale for plausibility (‘Makes no sense’ (1) to ‘Makes perfect sense’ (7)). They then rated each semantically-congruent sentence produced by the Mandarin-accented speaker for intelligibility on a seven-point scale (‘Impossible to understand’ (1) to ‘Very easy to understand’ (7)). Finally, to ensure that the foreign-accented speaker’s sentences were actually perceived as being foreign-accented, participants were asked to rate the overall degree of accentedness of the foreign-accented speaker on a seven-point scale (‘No foreign accent’ (1) to ‘Very strong foreign accent’ (7)). This accentedness rating was based on the sentences the listeners had already heard, i.e., the morphosyntactic and semantic sentences only, which did not contain any consonant clusters (beyond those caused by addition of the third person singular *-s*). This may have had the effect of minimising the apparent foreign-accentedness of these sentences. To ensure that the overall accent rating was representative of all sentences included in the task (i.e. both semantic and morphosyntactic sentences and fillers), participants then listened to ten consecutive filler sentences produced by the foreign-accented speaker and made a second accentedness rating based on these sentences. The overall accentedness rating was given by averaging ratings for the morphosyntactic and semantic sentences and the fillers.

In order to be included in the EEG task, semantically-congruent target sentences were required to receive a mean plausibility rating of five or greater, while semantically-incongruent sentences required a mean plausibility rating of three or less. Additionally, a mean intelligibility rating greater than 4.5 was used to signify that a given sentence was sufficiently intelligible to be included. Any sentences that did not achieve these ratings were discarded and replacement sentences were recorded using the same procedure as outlined above. These new sentences were then rated for plausibility and intelligibility in the same manner by a further ten participants aged 20 to 50 years (Mean = 32 years, SD = 10.3 years). The results yielded sufficient sentences with an acceptable rating to replace those discarded.

The accentedness of the foreign-accented speaker received a mean rating of 4.75 (SD = 0.91) based on the semantic and morphosyntactic sentences and 5.39 (SD = 0.99) for the fillers averaged across the two groups of participants (responses given by the two groups did not differ significantly from each other according to Wilcoxon rank sum tests; semantic and morphosyntactic sentences: U = 51.5, *p =* .93; filler sentences: U = 58.0, *p* = .53). The difference between the ratings given to the semantic and morphosyntactic sentences and the filler sentences across the two groups of participants was significant (Z = -3.31, *p* < .001) according to a Wilcoxon signed-rank test, demonstrating that the semantic and morphosyntactic sentences were indeed perceived as being less foreign-accented than the fillers. The sentences received an overall mean accentedness rating of 5.09 (SD = 1.00) across sentence types and both groups of participants, suggesting that the speaker’s accent was perceived as being moderate to strong overall.

### Procedure

During the test session, participants first completed an auditory digit span task (both forward and backward spans) to assess their working memory capacity [39], followed by a pre-task questionnaire. The questionnaire was designed to confirm that the participant fulfilled the criteria to participate in the study and to collect information to be used in the analysis of individual variability: the participant’s age and gender, and a measure of cognitive and affective empathy (the Empathy Quotient Short Form) [40].

**Fig 3:**
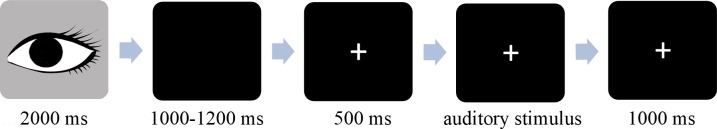
Schematic diagram of the trial structure.

EEG data were then collected while participants sat in a comfortable chair in front of a computer monitor in a sound-attenuated, electromagnetically-shielded room. During the task participants were presented with one of two lists of the 400 sentences via in-ear headphones. The sentences were presented in pseudorandomised order across eight blocks of 50 sentences each, with a practice block of ten additional filler sentences at the beginning of the experiment to familiarise participants with the task procedure. Each trial began with a cartoon image of an eye, presented on the screen for 2000 ms. Participants were instructed to blink whenever this image appeared on the screen. This was followed by a blank screen randomly varying in duration between 1000 and 1200 ms. A fixation cross then appeared on the screen for 500 ms before the onset of the auditory stimulus sentence and remained on the screen until 1000 ms after the completion of the sentence (see Fig 3).

Participants were presented with five comprehension questions per block at irregular intervals to ensure they were attending to the sentences (40 questions in total). Questions always referred to the sentence immediately before and responses were recorded using a button box. If participants scored below 34 out of 40 (85% accuracy), they were considered to have been insufficiently attentive during the task and were excluded. However, all participants performed at or near ceiling for comprehension accuracy, with a mean score of 39.5 out of 40 (98.7%). No participant scored below 37 out of 40 (92.5%). Therefore no participants were excluded for failing to attend to the task. Additionally, a short game of ‘Snake’ (Gremlin Industries, USA, 1976) controlled by the button box appeared five times per block for participants to play in order to maintain their attention throughout the experiment.

### Data acquisition

Continuous EEG data were recorded using Curry software (Version 7; Compumedics Ltd., USA) from 64 Ag/AgCl scalp electrodes mounted on an electrode cap (Easycap, Brainworks, GmbH) according to the International 10–10 system [41] (Fpz, Fz, FCz, Cz, CPz, Pz, Oz, Fp1/2, AF7/3/1/2/4/8, F7/5/3/1/2/4/6/8, FT7/8, FC5/3/1/2/4/6, T7/8, C5/3/1/2/4/6, M1/2, TP7/8, CP5/3/1/2/4/6, P7/5/3/1/2/4/6/8, PO7/5/3/4/6/8, O1/2). Ground was located at AFz. Electrooculographic activity was measured from electrodes placed above and below the right orbit and at the outer canthus of each eye. Electrode impedances were kept below 10kΩ. Electrical activity was recorded from both mastoids, with the left mastoid as the on-line reference. The EEG signal was digitised at a sampling rate of 1000 Hz and filtered with a .05–100 Hz bandpass filter using a Neuroscan SynAmps2 DC Amplifier (Compumedics Ltd., USA).

The EEG recording lasted between one and one and a half hours for each participant, including breaks. After the EEG recording, participants completed a post-task questionnaire in which they were asked to rate the strength of the foreign-accented speaker’s accent on a seven-point scale from ‘No foreign accent’ (1) to ‘Very strong foreign accent’ (7), and to identify the nationality of the foreign-accented speaker. In all, the testing session for each participant lasted approximately two and a half hours.

### Data processing

EEG data were processed in MATLAB (Version R2015a, The MathWorks Inc., Natick, MA, US) using the Fieldtrip toolbox (Version 20160719) [42]. Epochs of approximately 5500 ms were extracted from the continuous data stream (from 1000 ms before the presentation of the fixation cross to 1500 ms after the offset of the audio file for each trial). An independent component analysis was then performed on the data to remove artefacts caused by eye-blinks, saccades, and pulses [43]. The data were re-referenced off-line to the average of the mastoids. A baseline correction was then performed, with the 200 ms before the time-locking point of the ERP as the baseline period. (Before baseline correction, a cluster-based permutation test [44] was performed on the data, which showed no significant differences between conditions during the baseline period, confirming its suitability). A zero-phase fourth-order Butterworth IIR low-pass filter of 35 Hz was applied to the dataset, with a 12 dB per octave slope. Epochs were then redefined as the period from the beginning of the baseline period to 1500 ms after the time-locking point (the onset of the object noun).

Trials with remaining artefacts, defined as those epochs in which the amplitude variance was greater than 1000 μV and/or the maximum z value was greater than 4, were then removed from the data set. In total, 227 of the 3000 trials for this study were rejected (7.6%). These were evenly distributed across sentence types. Once trials containing artefacts had been rejected, the data were averaged within sentence types for each participant, then a grand average for each sentence type was calculated across participants.

### Statistical analysis

Cluster-based permutation tests (CBPTs) [44] were used to compare neural responses between conditions. To conduct a CBPT, a t-test is first performed on the amplitude values of the two waveforms to be compared at each individual sampling point. Sampling points at which the difference between the waveforms is found to be significant (*p* < 0.05, two-tailed) are then formed into clusters based on both temporal and spatial adjacency, and the polarity of the effect (i.e. t-values associated with sampling points in a single cluster must be either all positive or all negative). Each electrode has a mean of 6.27 ‘neighbour’ electrodes (range = 2–8) which are considered spatially adjacent. Cluster-level statistics are then calculated by adding together all t-values within the cluster. The largest of these cluster-level statistics are then tested for significance against clusters of samples selected using 1000 random partitions. CBPTs are therefore able to provide information about the polarity, amplitude, latency (duration from effect onset to offset), onset latency (time point of the effect onset), and topographical distribution of any ERP effect. They are also recommended by Luck and Gaspelin [45] as a method which minimises the possibility of Type I error in ERP analysis, compared to the traditional approach of using pre-defined time-windows and multi-level ANOVAs for analysis.

Three CBPTs were performed on the data across the 1000 ms following the time-locking point of the ERP. The first compared neural responses to semantically congruent and incongruent sentences produced by the native speaker to test if an N400 effect was elicited by the native speaker’s semantic violations. The second CBPT compared semantically congruent and incongruent sentences produced by the foreign-accented speaker to test if an N400 effect was elicited by this speaker’s violations. Finally, neural responses to semantically incongruent sentences for each speaker were subtracted from responses to that speaker’s semantically congruent sentences. These difference waves were then compared using a third CBPT to examine whether the characteristics of the N400 effects differed between the two speaker conditions, i.e. an interaction between Speaker and Congruity.

#### Individual variability

A linear mixed-effects model was then used to investigate which factors, if any, might have been responsible for individual variation in participants’ neural responses. The dependent measure was the mean ERP component amplitude of each of the four conditions (see Table 1) for each participant, averaged across the time period in which the significant effects in the two speaker conditions temporally overlapped. The individual-variability factors included in the model were the participants’ Age, Gender, Working memory, Empathy, Accentedness rating and Accent familiarity. The indices used for these factors were as follows: Scores from the auditory digit span task (combined forward and backward spans) were converted to an index of working memory according to the Wechsler Adult Intelligence Scale [39]. Scores calculated from the Empathy Quotient Short Form according to the method laid out by Wakabayashi and colleagues [40] were used to provide an index of participant empathy. Participants’ post-task ratings of the foreign-accentedness of the speech they heard during the EEG task were used to represent how strong the participants perceived the Mandarin-accented speaker’s speech to be (Accentedness rating). Participants’ identification of the foreign-accented speaker’s nationality in the post-task questionnaire was coded as being either correct or incorrect and was used as a measure of Accent familiarity (i.e., participants who could correctly identify the foreign-accented speaker’s nationality as being Chinese were taken as being more familiar with the accent in question, cf. [6]). All continuous factors (i.e. Age, Empathy, Working memory and Accentedness rating) were centred by subtracting each observation from the mean value for that factor. All categorical factors (i.e. Gender, Speaker, Congruity, and Accent familiarity) were coded using Deviation coding (female = -1, male = 1, native-accent = 1, foreign-accent = -1, congruous = -1, incongruous = 1, correct identification of accent = 1, incorrect identification of accent = -1).

The model was fit using the *lmer()* function of the lme4 package (Version 1.1.17) [46] in R (Version 3.5.0; R Core Team, 2016). First, a full model of component amplitude was created, including interactions between Congruity and Speaker and each of the individual-variability factors, by-participant random intercepts and by-speaker random slopes. The syntax of the full model was: lmer(amplitude ~ congruity * speaker * (age + gender + empathy + workingMemory + accentednessRating + accentFamiliarity) + (speaker | participant)). Model comparisons were then performed to determine the most parsimonious model, according to the Akaike information criterion (AIC). Significance tests were performed on the most parsimonious model using the lmerTest package (Version 3.0.1) [47]. Post-hoc tests on significant interactions were conducted using the *lsmeans()* function of the lsmeans package (Version 2.27.62) [48] in R. Holm-Bonferroni correction was used to compensate for multiple pairwise comparisons. The Welch-Satterthwaite equation was used to calculate approximated degrees of freedom.

## Results

**Fig 4:**
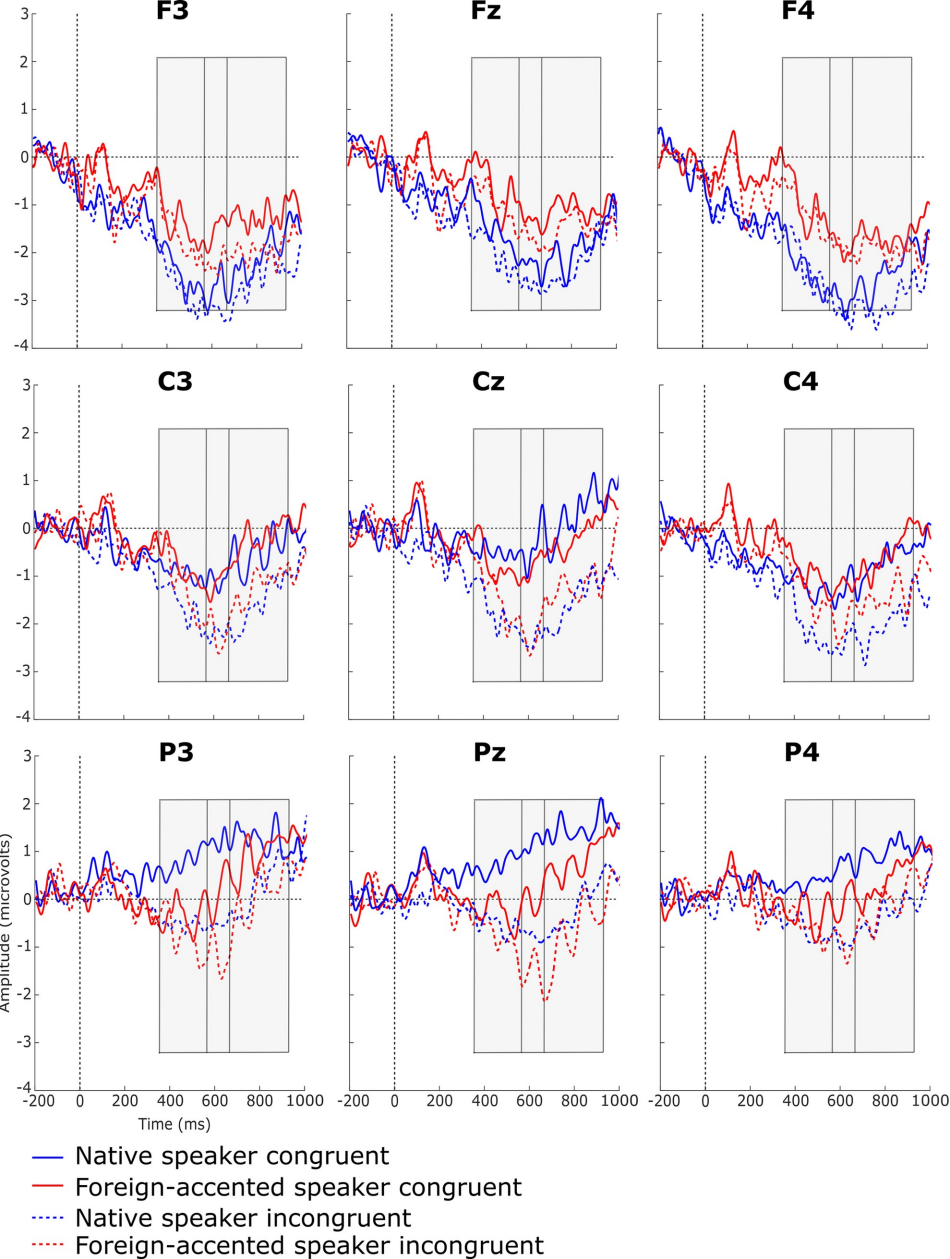
Grand average waveforms of semantically congruent and incongruent conditions for the native-speaker and foreign-accented-speaker conditions. Negativity is plotted downwards. Grey boxes represent the time periods of the significant differences between congruent and incongruent sentences as shown by cluster-based permutation tests.

Fig 4 shows the grand average waveforms for the semantically congruent and incongruent sentences produced by the native and foreign-accented speakers. Increased negativity can be observed in responses to the semantically incongruent sentences compared to the congruent sentences for both speakers, most notably at the central and parietal electrodes.

**Fig 5:**
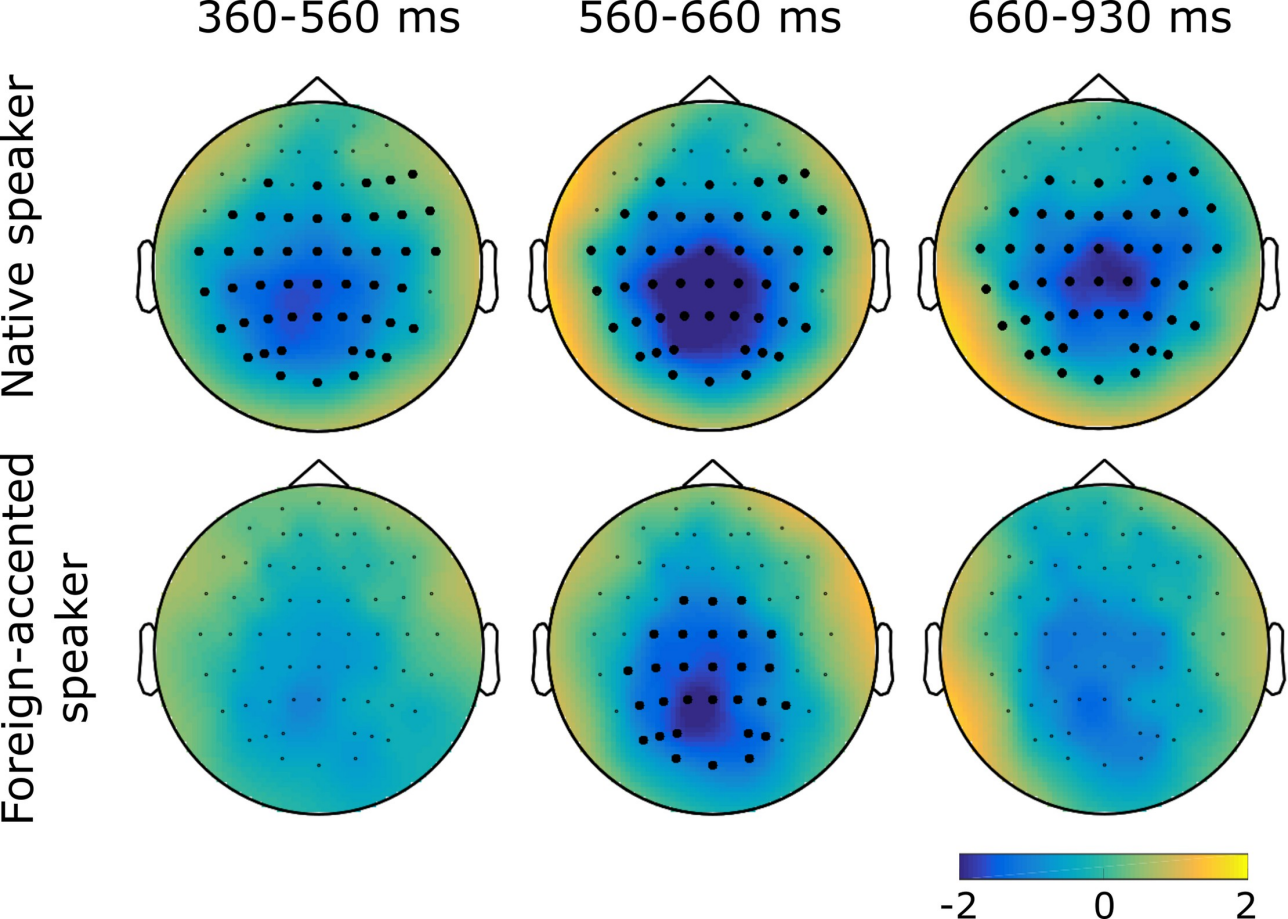
Topographical maps showing the amplitude difference between semantically incongruent and congruent conditions. Native-speaker condition in the top row and foreign-accented-speaker condition in the bottom row. Centre column shows the time period in which the effects temporally overlap. Topographies interpolated from 64 electrodes. Electrodes contributing to the significant effects are shown as filled black circles.

**Fig 6:**
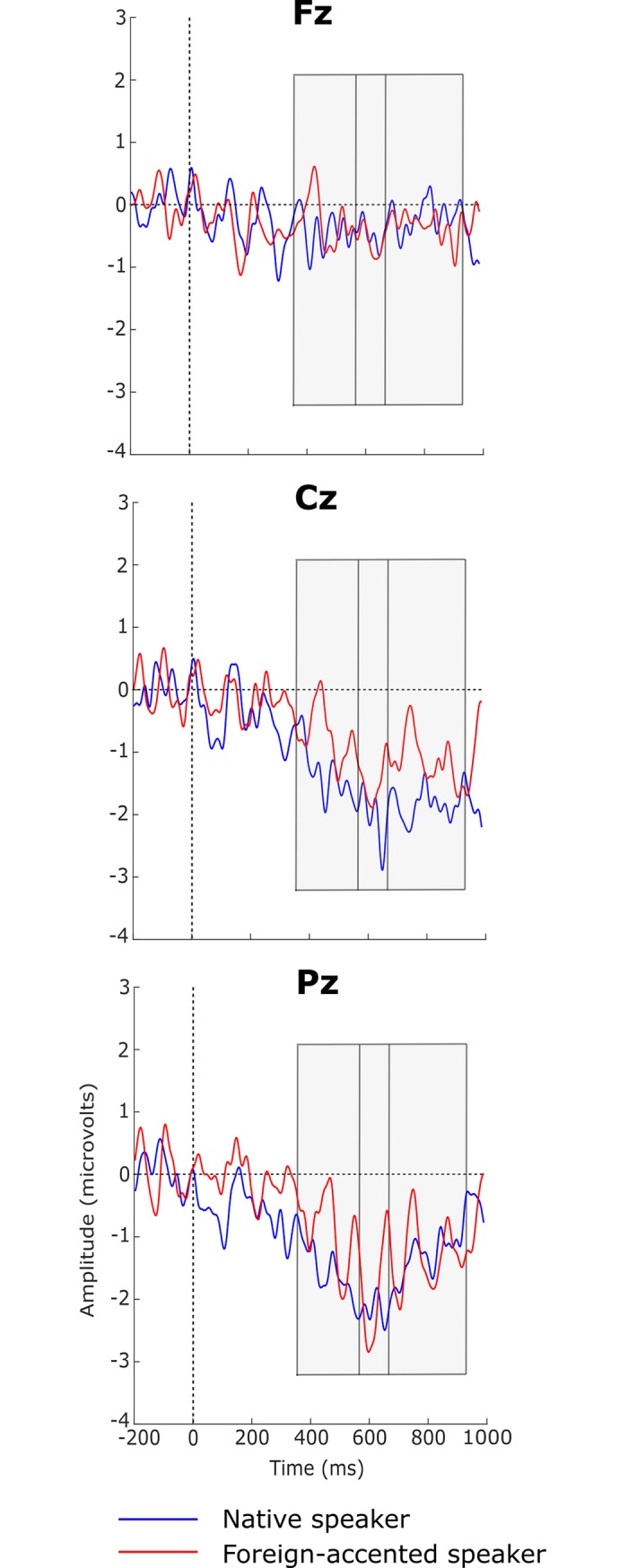
Grand average difference waves between the congruent and incongruent conditions in native- and foreign-accented-speaker conditions. Negativity is plotted downwards.

The first CBPT, comparing neural responses to semantically congruent and incongruent sentences produced by the native speaker, revealed a significant difference between the two conditions (*p* < .001): Compared to the semantically congruent sentences, semantically incongruent sentences elicited a more negative response with a broad distribution between 364 and 931 ms after the onset of the target word. The second CBPT compared neural responses to semantically congruent and incongruent sentences produced by the foreign-accented speaker, again revealing a significant difference between the two conditions (*p* = .03): Once again, the semantically incongruent sentences showed a more negative response, this time with a predominantly parietal distribution between 564 and 657 ms after the onset of the target word (see Fig 5 for topographical maps of these effects). The third CBPT examined whether the difference between semantically congruent and incongruent sentences for the native speaker differed from that of the foreign-accented speaker, but no significant difference was found (*p* ≥ .22; see Fig 6).

Mean component amplitudes for each participant per condition were then entered into the linear mixed-effects model. Mean amplitudes were calculated across the 560 to 660 ms time window, as this was the time period in which the two significant ERP effects overlapped (rounded to the nearest 10 ms). Model comparisons revealed that the most parsimonious model included interactions of Congruity and Speaker with Age, Working memory and Accent familiarity, the main effect of Accentedness rating, by-participant random intercepts and by-speaker random slopes. The syntax of the final model was: lmer(amplitude ~ congruity * speaker * (age + workingMemory + accentFamiliarity) + accentednessRating + (speaker | participant)). This model showed significant main effects of Congruity (β = -0.71, SE = 0.10, *p* < .001), Age (β = -0.09, SE = 0.03, *p* = .004) and Accentedness rating (β = 0.35, SE = 0.13, *p* = .01): Component amplitude was more negative for incongruous than congruous sentences and became more negative with increasing age, but became more positive with increasing accentedness rating. There was also a significant two-way interaction between Congruity and Working memory (β = -0.06, SE = 0.02, *p* = .006), and significant three-way interactions between Congruity, Speaker and Age (β = 0.05, SE = 0.02, *p* = .02), and Congruity, Speaker and Accent familiarity (β = 0.26, SE = 0.11, *p* = .03). No other main effects or interactions reached significance (all *ps* ≥ .07).

**Fig 7:**
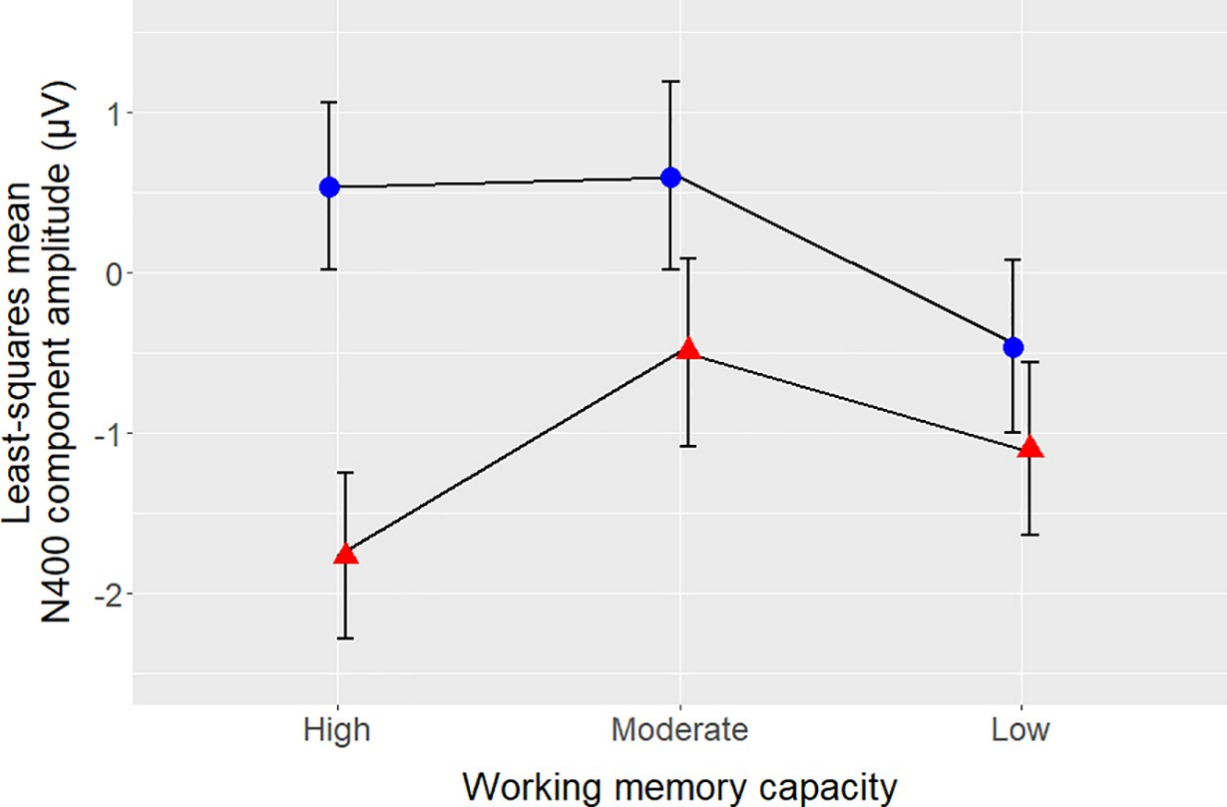
The relationship between N400 component amplitude and working memory capacity. Least-squares mean amplitude values for congruous sentences are shown as blue circles, least-squares mean amplitude values for incongruous sentences are shown as red triangles. Error bars show 95% confidence intervals. High-, moderate- and low-working-memory-capacity groups were defined by digit span scores as used in the post-hoc analysis.

To further investigate the interaction between Congruity and Working memory, participants were divided into three groups of approximately equal size according to their digit span score (high: score of 20–27, n = 11; middle: score of 15–19, n = 10; low: score of 9–14, n = 9). Pairwise comparisons were performed to determine whether the effect of Congruity was present for each of these groups. Responses to incongruent sentences were significantly more negative than responses to the congruent sentences for both the high- (*t*(50) = 6.88, *p* < .001) and middle-scoring (*t*(50) = 2.88, *p* = .006) groups, but not the low-scoring group (*t*(50) = 1.83, *p* = .07; Fig 7).

**Fig 8:**
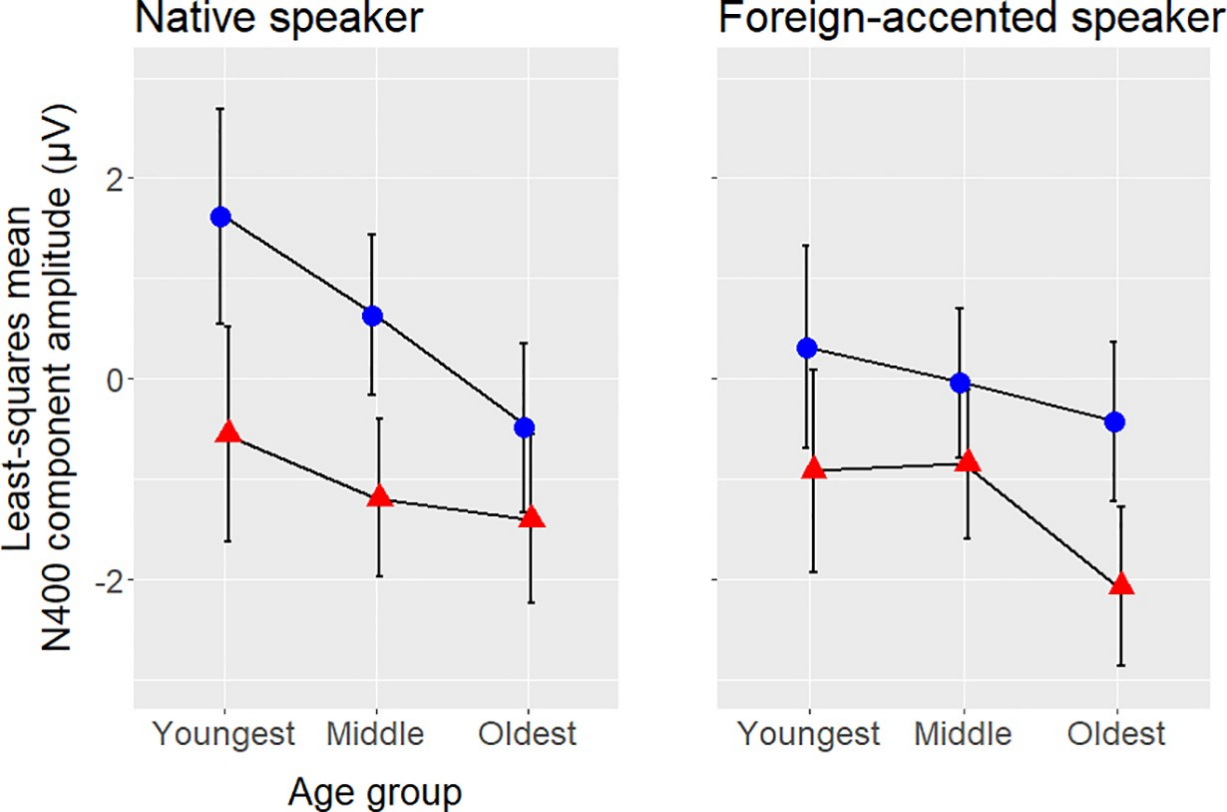
The relationship between N400 component amplitude and age for each speaker condition. Least-squares mean amplitude values for congruous sentences are shown as blue circles, least-squares mean amplitude values for incongruous sentences are shown as red triangles. Error bars show 95% confidence intervals. Youngest, middle and oldest age groups were defined as in the post-hoc analysis.

Similarly, to investigate the interaction between Congruity, Speaker and Age, participants were divided into three groups of approximately equal size according to their age (youngest: 18 years, n = 9; middle: 19–24 years, n = 11; oldest: 25–33 years, n = 10). Pairwise comparisons across Congruity and Speaker showed that when listening to the native speaker, responses to incongruent sentences were significantly more negative than responses to congruent sentences for the youngest (*t*(50) = 3.40, *p* = .001) and middle (*t*(50) = 3.81, *p* < .001) age groups, but not the oldest (*t*(50) = 1.79, *p* = .08). While listening to the foreign-accented speaker, only the oldest group showed significantly more negative responses to the incongruent than congruent sentences (*t*(50) = 3.23, *p* = .002), not the middle (*t*(50) = 1.69, *p* = .10) or youngest (*t*(50) = 1.92, *p* = .06) age groups (see Fig 8).

**Fig 9:**
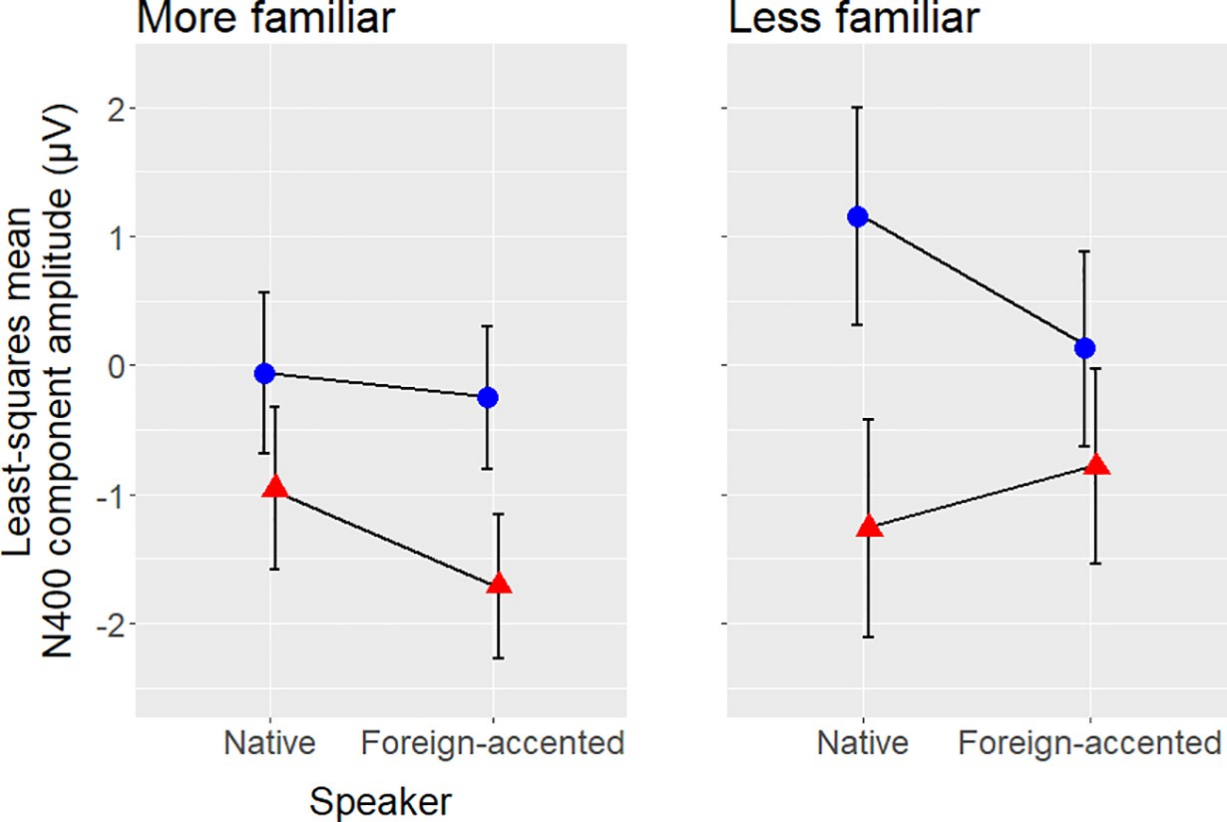
The relationship between N400 amplitude and accent familiarity. Least-squares mean amplitude values for congruous sentences are shown as blue circles, least-squares mean amplitude values for incongruous sentences are shown as red triangles. Error bars show 95% confidence intervals.

Finally, pairwise comparisons to investigate the interaction between Congruity, Speaker and Accent familiarity showed that responses to incongruent sentences were significantly more negative than responses to congruent sentences regardless of speaker for the more familiar listeners (i.e. those who had correctly identified the foreign-accented speaker’s accent; native speaker: *t*(52) = 2.46, *p* = .02; foreign-accented speaker: *t*(52) = 4.02, *p* < .001). The less familiar listeners only showed this pattern for sentences produced by the native speaker (*t*(52) = 4.92, *p* < .001), and did not show a significant difference in responses to the congruent and incongruent sentences produced by the foreign-accented speaker (*t*(52) = 1.86, *p* = .07; see Fig 9).

## Discussion

### Responses to semantic violations in native vs. foreign-accented speech

The first aim of the current study was to test whether semantic violations in foreign-accented speech elicited different ERP effects from those in native speech in a linguistic environment where foreign-accented speech was relatively common, using a method that could account for all characteristics of an ERP component. We hypothesised that the same ERP effect, an N400 effect, would be found for both speakers’ semantic violations, but that these N400 effects would differ in terms of their amplitude, topography, and/or onset latency. Semantically-incongruent sentences did elicit a significantly larger negativity than semantically-congruent sentences in both speaker conditions. The larger negativity in the native-speaker condition showed a broad distribution with a centro-parietal maximum while the larger negativity in the foreign-accented-speaker condition showed a centro-parietal distribution (see Fig 5). These differences are interpreted as N400 effects, motivated by their polarity and scalp distribution. This supports our hypothesis that an N400 effect would be present for semantic violations produced by both speakers.

The CBPTs performed separately for the two speakers appeared to show differences in topography, component latency and onset latency between the two speaker conditions: The N400 for foreign-accented speech seemed less broadly distributed (see Fig 5 above), shorter in latency (93 vs 567 ms), and later in onset latency (564 vs 364 ms) than that for native speech. To test whether these differences were significant, a CBPT was conducted on the difference waves of the two speaker conditions, but it revealed no significant differences. That is, any apparent difference between the two N400s did not reach statistical significance. This seeming inconsistency may be due to the sensitivity of CBPTs to signal-to-noise ratio. Data in the foreign-accented-speaker condition could potentially be noisier than those of the native-speaker condition (for example, if inter-listener variability in responses was greater for foreign-accented than native speech), resulting in an apparently smaller effect for the foreign-accented speaker when individual CBPTs were performed. However, without a suitable metric to quantify data noisiness across conditions, this is a speculative explanation.

These results contradict our hypothesis that the N400s observed for violations produced by the two speakers would differ in their amplitude, topography and/or onset latency, as no differences on any dimension were found. This also goes against the results of other studies comparing neural responses to semantic violations in native and foreign-accented speech, which all found differences between the two speaker conditions [1,5,6]. There are two potential explanations for the lack of a difference in the current study: the statistical methods used, and the linguistic environment in which the study was conducted.

The first potential explanation for the lack of difference between the N400s for the two speakers relates to the use of CBPTs for statistical analysis. The CBPT is a conservative statistical method which greatly minimises the risk of finding false positive results in comparison to the often-employed method of analysing ERP data with multiple ANOVAs across pre-defined time windows [45]. As pre-defined time windows or time-windows based on visual inspection of the data and multiple ANOVAs and post-hoc tests were used in each of the studies finding significant differences between N400s for native and foreign-accented speech [1,5,6], it is possible that these less-conservative statistical methods were responsible for the effects found in these studies, as the possibility of Type I error was greatly inflated. The inconsistent nature of the differences found between N400 effects in the previous studies lends some support to the proposal that the effects may be spurious. Furthermore, according to simulations carried out by Luck and Gaspelin [45], the probability of at least one Type I error occurring in an ERP analysis based on factorial ANOVAs may be as high as 96%, depending on study design. Alternatively, it is possible that the linguistic environment that participants in the current study were exposed to may have been responsible for the lack of effect found. Evidence for this comes from the analysis of individual variability, discussed below.

### Individual variability in N400 amplitude

The analysis of individual variability in listeners’ neural responses revealed several factors which significantly influenced N400 effect amplitude. Note that, as we were interested in factors that affect *effect* amplitude, rather than *component* amplitude, we will focus here only on factors which interacted with Congruity. Three factors were found to influence N400 component amplitude depending on the congruity of the sentence: familiarity, working memory and age.

#### Familiarity

Listeners showed a significant N400 effect for semantic violations produced by the native speaker (i.e., responses elicited by incongruent sentences were significantly more negative than for congruent sentences), regardless of their level of familiarity with the Mandarin accent used in the study. However, when listening to the foreign-accented speaker, only those listeners who were more familiar with the accent showed a significant N400 effect (see Fig 9 above, S1 and S2 Figs). This contradicted our hypothesis regarding the effect of familiarity on N400 amplitude: We had anticipated that the foreign-accented speaker’s errors might elicit a *smaller* effect amplitude for more familiar than less familiar listeners, as they might be more likely to anticipate errors from the accented speaker and thus be more lenient towards them. Instead we found a *greater* effect amplitude for more familiar listeners, with the less familiar listeners failing to show a significant effect at all. It seems that listeners who were more familiar with the foreign-accented speech were more likely to process sentences produced by the native and foreign-accented speakers the same way, without affording the foreign-accented speech any ‘special treatment’. Less familiar listeners, on the other hand, may have been lenient towards errors produced by the foreign-accented speaker, resulting in the lack of effect for that condition, due to their lack of knowledge about the types of errors foreign-accented speakers typically make. Alternatively, lack of familiarity with foreign-accented speech may have caused these listeners to divert processing resources to other aspects of sentence processing to aid their understanding, resulting in a superficial level of semantic processing (cf. ‘good enough’ processing [49]).

This familiarity effect may explain why no group-level difference was found between N400 effects for native and foreign-accented speakers in the current study. Taken alone, the pattern of results observed for the group of participants with less foreign-accent familiarity more closely resembles the results found in existing studies comparing the N400 effect elicited by native and foreign-accented speech [1,5,6], in that the characteristics of the two N400 effects differ. Thus, it could be the case that previous studies found overall differences between the two N400s as the majority of the participants in their studies had minimal foreign-accent experience due to the relatively small immigrant populations of their respective places of residence, as described previously. Conversely, the majority of participants in the current study had greater foreign-accent experience (i.e. 19 out of 30 (63%) correctly identified the accent) and did not show any difference in N400 effect characteristics between speakers, thus resulting in a lack of overall difference at the group level.

As discussed above, the results of previous studies of semantic processing in foreign-accented speech all showed a difference in the N400 effect between native and foreign-accented speech but were conflicting in terms of which component characteristics differed [1,5,6]. While the results observed for the less-familiar group in our study are more consistent with those found in previous studies in that there is a difference between responses to native and foreign-accented speech, they differ from other studies in terms of what the difference is. The difference between speaker conditions for the less-familiar listeners in the current study manifested as a smaller N400 effect amplitude for foreign-accented than for native speech (to the extent that the N400 effect for accented speech was not significant). This contrasts with the lack of amplitude difference found in [1] and the greater effect amplitude found in [5]. We also did not observe the topographical differences found in [1,5] or the onset latency difference found in [6]. It is unclear why, even among the less-familiar listeners in the current study, the results should remain incongruent with previous studies. This finding highlights the ongoing lack of consensus as to the effect of a foreign accent on the N400 effect, specifically among less-familiar listeners.

#### Working memory

As hypothesised, increased working memory capacity was associated with a greater difference between responses to congruous vs. incongruous sentences (i.e., increased N400 effect amplitude), regardless of speaker: Significant N400 effects were found for listeners with moderate to high digit span scores, but not listeners with low scores. This finding is consistent with that of Van Petten et al. [19], who found progressively smaller N400s in native speech processing with decreasing working memory scores, where listeners with the lowest working memory scores showed no N400 at all. Van Petten and colleagues suggested that listeners with lower working-memory capacity are less efficient at taking sentential context into account when processing semantic information. We adopt this explanation for the working memory effect found in the current study as well.

However, the association between N400 amplitude and working memory in the current study differs from the results of other studies of both native and foreign-accented speech processing. Regarding native speech, Nakano, Saron and Swaab [50] found no difference in N400 amplitude for semantic violations across high- and low- working memory listeners: They found a relationship between working memory and N400 amplitude for animacy violations, but not for semantic violations in which animacy was not violated. However, this lack of effect could potentially be attributed to the methodological choice of performing a median split to group participants into high- and low-working-memory groups. Using two rather than three groups may have masked a more subtle gradient relationship between N400 amplitude and working memory in the no-animacy-violation condition. On the other hand, Boudewyn and colleagues [18] found that the topographical distribution, rather than the amplitude of the N400 effect elicited by semantic incongruities, was modulated by working memory capacity. However, as the design of the individual variability analysis in the current study accounts for variability in effect amplitude only, rather than variability in topographical distribution, the results of the current study and [18] are not comparable.

Regarding foreign-accented speech, Grey and Van Hell [6] found no significant relationship between working memory capacity and N400 amplitude, in contrast to the current study. It is somewhat unclear why these two studies found different results; a possible reason could be due to methodological differences. Grey and Van Hell measured working memory capacity using an O-span (complex memory span) task [51], while the current study used a combination of forward and backward digit spans. The combined digit span measure may incorporate the phonological loop subcomponent of working memory to a greater extent than the O-span [52], which relies more on the central executive component of working memory, meaning that these measurements of working memory capacity are not tapping exactly the same phenomena. It may be that the phonological loop plays a more important role in semantic processing than the central executive component, and thus a relationship to N400 amplitude was found only when a measure that heavily relied on the phonological loop was used to measure working memory capacity. Also, fewer data points were available for the correlational analysis carried out in [6] than the current study as in [6] variability in responses to native and foreign-accented speech were investigated separately rather than in the same model. This smaller amount of data may have contributed to the lack of effect found in [6].

We had also hypothesised that any effect of working memory on N400 amplitude would be exaggerated in the foreign-accented speech condition, due to the increased use of working-memory resources in processing foreign-accented speech [28]. However, we found no evidence for this, as there was no significant interaction between Congruity, Working memory, and Speaker.

#### Age

An interaction was present between congruity, speaker and participant age (see Fig 8): Younger listeners (aged 18 to 24) showed a significant N400 effect in response to violations produced by the native speaker but not the foreign-accented speaker, while older listeners (aged 25 to 33) showed the reverse pattern. This age effect was unexpected, as the study was not designed to examine effects of age and thus included only a narrow age range. While age effects on N400 amplitude have previously been observed for native speech, they typically occur across larger age ranges than in the current study (i.e. elderly adults vs. young adults) [53]. The effect of age, like the effect of familiarity discussed above, may also have contributed towards the lack of difference found between responses to the native and foreign-accented speakers at the group level. If younger listeners showed greater N400 effects in response to native than foreign-accented speech and older listeners showed the reverse, these differences may have cancelled out to some extent, contributing to the equivalent N400 effects in the two speaker conditions.

However, it remains unclear why the effect of age should differ between the native and foreign-accented speech, rather than remaining consistent across speakers. One potential difference between the younger and older listeners that could explain an age effect is the increased likelihood of high-frequency hearing loss among older listeners, despite self-reported normal hearing, which may affect signal audibility. However, while this may affect N400 amplitude, we would expect it to have an equivalent, rather than opposite, effect in the two speaker conditions. Similarly, older listeners may make less use of surrounding linguistic context to facilitate semantic integration or semantic-memory retrieval [53–55], again affecting their N400 effect amplitude, though we would again expect this to have a similar effect across native and foreign-accented speech.

Alternatively, older and younger listeners may differ in their degree of exposure to foreign-accented speech: Younger listeners are likely to have encountered Mandarin-accented speech at a younger age than older listeners, due to the relative recency of large-scale migration from China to Australia, and thus may be more familiar with it. However, independent effects of familiarity, controlling for age, and age, controlling for familiarity, were found in the analysis of individual variability, suggesting that the age effect cannot be entirely attributed to listeners’ familiarity with foreign-accented speech. It may be the case that, had participants with a broader age range been included in the study, a more conclusive and interpretable pattern of results may have become evident. Further research is thus required to make a strong claim for the effect of age on neural responses to semantic violations.

### Implications

As mentioned previously, foreign-accented speech is commonly encountered in everyday life, and thus must be accounted for by models of sentence processing. However, the current study has demonstrated that the impact of a foreign accent on semantic processing is not an all-or-nothing phenomenon: Gradient effects of foreign-accented speech may be observed, mediated by the characteristics of individual listeners such as familiarity, working memory capacity and, potentially, age. This suggests that models of semantic processing, and sentence processing more broadly, must not only account for processing changes due to characteristics of the *speaker* (e.g., foreign-accentedness), but also individual processing differences due to characteristics of the *listener*, and interactions between these factors. From this perspective, the results of the current study provide support for more interactive models of sentence processing [24–27]. These models can account for the integration of multiple sources of not only linguistic information during sentence processing, but also non-linguistic information, including socio-indexical information about the characteristics of the speaker, as is the case here.

By conducting the current study in a linguistic environment where foreign-accentedness is widespread, we also observed a different pattern of results to previous studies conducted in locations were foreign-accentedness was less common [1,5,6]. This provides some evidence that the ambient linguistic environment can have a considerable impact on the results obtained in a sentence processing study using ERPs. This suggests that the linguistic environment must be taken into account in future studies of sentence processing.

A direction for future research also arises from a potential limitation of the current study, where only a single native speaker and a single foreign-accented speaker provided the stimuli. While this approach was chosen to increase the consistency of the stimuli, minimise unnecessary variability in neural responses due to speaker-specific effects, and align with the procedure used in previous studies [1,6], the result is that we cannot definitively state that the difference in responses to the two speakers found among the less-familiar listeners was due to the foreign-accentedness of the speaker per se. It is possible that some idiosyncratic property of the speech of one or both speakers is in fact responsible for the effect. We believe this is unlikely, as the two speakers were chosen for their canonical native Australian and Mandarin-accented speech respectively, however further research involving stimuli produced by a greater number of native and foreign-accented speakers would be valuable to provide supporting evidence for our findings.

## Conclusion

This study aimed to compare the N400 effects elicited by listening to semantic violations in native vs. foreign-accented speech in a linguistic environment where listeners were likely to be familiar with foreign-accented speech. Semantic processing in the native and foreign-accented speaker conditions elicited N400 effects that did not differ in amplitude, topography, latency or onset latency at the group level. However, a significant difference in N400 amplitude between speaker conditions was found among the subset of listeners who were less familiar with accented speech. Participants’ working memory capacity, and potentially age, were also found to modulate N400 amplitude. These findings suggest that participants’ linguistic environment can have a large impact on how they process native vs. foreign accented speech, highlighting the susceptibility of the N400 effect to variation from a range of sources relating to the characteristics of both the listener and the speaker.

## Supporting information

S1 Table Target stimuli. (DOCX)

S1 Fig Grand average waveforms for semantically congruent and incongruent conditions for the native-speaker and foreign-accented-speaker conditions shown by participants with greater familiarity with foreign-accented speech. Negativity is plotted downwards. Grey boxes represent the time periods of the significant differences between congruent and incongruent sentences as shown by cluster-based permutation tests.
(TIF)

S2 Fig Grand average waveforms for semantically congruent and incongruent conditions for the native-speaker and foreign-accented-speaker conditions shown by participants with less familiarity with foreign-accented speech. Negativity is plotted downwards. Grey boxes represent the time periods of the significant differences between congruent and incongruent sentences as shown by cluster-based permutation tests.
(TIF)

## References

[1] Hanulíková A, van Alphen PM, van Goch MM, Weber A. When One Person’s Mistake Is Another’s Standard Usage: The Effect of Foreign Accent on Syntactic Processing J Cogn Neurosci. MIT Press; 2012;24: 878–887. 10.1162/jocn_a_00103 21812565 10.1162/jocn_a_00103

[2] Simons GF, Fennig CD. Ethnologue [Internet]. 20th ed Dallas, Texas: SIL International; 2017 Available: https://www.ethnologue.com/

[3] Piske T, MacKay IRA, Flege JE. Factors affecting degree of foreign accent in an L2: a review. J Phon. 2001;29: 191–215. 10.1006/jpho.2001.0134

[4] Sydney LocalStats Australia [Internet]. [cited 17 Aug 2017]. Available: http://localstats.com.au/demographics/nsw/sydney

[5] Romero-Rivas C, Martin CD, Costa A. Processing changes when listening to foreign-accented speech. Front Hum Neurosci. 2015;9 10.3389/fnhum.2015.00167 25859209 10.3389/fnhum.2015.00167 PMC4373278

[6] Grey S, van Hell JG. Foreign-accented speaker identity affects neural correlates of language comprehension. J Neurolinguistics. Pergamon; 2017;42: 93–108. 10.1016/J.JNEUROLING.2016.12.001

[7] Luck SJ. An Introduction to the Event-Related Potential Technique, Second Edition Second. Cambridge, Massachusetts: MIT Press; 2014.

[8] Kutas M, Hillyard SA. Reading Senseless Sentences—Brain Potentials Reflect Semantic Incongruity. Science (80-). 1980;207: 203–205. 7350657 10.1126/science.7350657

[9] Kutas M, Federmeier KD. Thirty years and counting: finding meaning in the N400 component of the event-related brain potential (ERP). Annu Rev Psychol. 2011;62: 621–647. 10.1146/annurev.psych.093008.131123 20809790 10.1146/annurev.psych.093008.131123 PMC4052444

[10] van de Meerendonk N, Kolk HHJ, Vissers CT, Chwilla DJ. Monitoring in language perception: mild and strong conflicts elicit different ERP patterns. J Cogn Neurosci. 2009/02/10. 2010;22: 67–82. 10.1162/jocn.2008.21170 19199401 10.1162/jocn.2008.21170

[11] Romero-Rivas C, Martin CD, Costa A. Foreign-accented speech modulates linguistic anticipatory processes. Neuropsychologia. 2016;85:245–55. 10.1016/j.neuropsychologia.2016.03.022 27020137 10.1016/j.neuropsychologia.2016.03.022

[12] Goslin J, Duffy H, Floccia C. An ERP investigation of regional and foreign accent processing. Brain Lang. 2012;122: 92–102. 10.1016/j.bandl.2012.04.017 22694999 10.1016/j.bandl.2012.04.017

[13] Floccia C, Butler J, Goslin J, Ellis L. Regional and foreign accent processing in English: Can listeners adapt? J Psycholinguist Res. 2009;38: 379–412. 10.1007/s10936-008-9097-8 19117134 10.1007/s10936-008-9097-8

[14] U.S. Census Bureau QuickFacts: Pennsylvania [Internet]. 2016 [cited 14 Dec 2017]. Available: https://www.census.gov/quickfacts/fact/map/PA/POP645216#viewtop

[15] Brinkoff T. City Population [Internet]. [cited 17 Aug 2017]. Available: https://www.citypopulation.de/php/netherlands-gelderland.php?cityid=201

[16] Barcelona Population [Internet]. [cited 17 Aug 2017]. Available: http://worldpopulationreview.com/world-cities/barcelona-population/

[17] Bornkessel-Schlesewsky I, Philipp M, Alday PM, Kretzschmar F, Grewe T, Gumpert M, et al Age-related changes in predictive capacity versus internal model adaptability: Electrophysiological evidence that individual differences outweigh effects of age. Front Aging Neurosci. 2015;7 10.3389/fnagi.2015.00217 26648865 10.3389/fnagi.2015.00217 PMC4663277

[18] Boudewyn MA, Long DL, Swaab TY. Effects of working memory span on processing of lexical associations and congruence in spoken discourse. Front Psychol. 2013;4: 60 10.3389/fpsyg.2013.00060 23407753 10.3389/fpsyg.2013.00060 PMC3570772

[19] Van Petten C, Weckerly J, Mclsaac HK, Kutas M. Working memory capacity dissociates lexical and sentential contact effects. Psychol Sci. 1997;8: 238–42. 10.1111/j.1467-9280.1997.tb00418.x

[20] van den Brink D, Van berkum JJA, Bastiaansen MCM, Tesink CMJY, Kos M, Buitelaar JK, et al Empathy matters: ERP evidence for inter-individual differences in social language processing. Soc Cogn Affect Neurosci. 2012;7: 173–183. 10.1093/scan/nsq094 21148175 10.1093/scan/nsq094 PMC3277364

[21] Baese-Berk M, Bent T, Borrie S, McKee M. Individual differences in perception of unfamiliar speech. In Proceedings of the 18th International Congress of Phonetic Sciences, ed The Scottish Consortium for ICPhS 2015 (pp. 0460–1).

[22] Ingvalson EM, Lansford KL, Fedorova V, Fernandez G. Cognitive factors as predictors of accented speech perception for younger and older adults. J Acoust Soc Am. 2017;141(6):4652–9. 10.1121/1.4986930 28679239 10.1121/1.4986930

[23] Friederici AD. The brain basis of language processing: from structure to function. Physiol Rev. 2011 10;91(4):1357–92. 10.1152/physrev.00006.2011 22013214 10.1152/physrev.00006.2011

[24] Bornkessel-Schlesewsky I, Schlesewsky M. Reconciling time, space and function: A new dorsal-ventral stream model of sentence comprehension. Brain Lang. 2013;125: 60–76. 10.1016/j.bandl.2013.01.010 23454075 10.1016/j.bandl.2013.01.010

[25] Pickering MJ, Garrod S. An integrated theory of language production and comprehension Behav Brain Sci. Cambridge University Press; 2013;36: 329–47. 10.1017/S0140525X12001495 23789620 10.1017/S0140525X12001495

[26] Hagoort P. MUC (Memory, Unification, Control) In: Hickok G, Small SL, editors. Neurobiology of Language. London: Academic Press; 2016 pp. 339–347. 10.1016/B978-0-12-407794-2.00028–6

[27] Martin AE. Language Processing as Cue Integration: Grounding the Psychology of Language in Perception and Neurophysiology [Internet]. Frontiers in Psychology. 2016 p. 120 Available: http://journal.frontiersin.org/article/10.3389/fpsyg.2016.00120 10.3389/fpsyg.2016.00120 26909051 10.3389/fpsyg.2016.00120 PMC4754405

[28] Van Engen KJ, Peelle JE. Listening effort and accented speech. Front Hum Neurosci. 2014;8 10.3389/fnhum.2014.00577 25140140 10.3389/fnhum.2014.00577 PMC4122174

[29] Holt R, Kung C, Schmidt E, Demuth K. Neural responses to morphosyntactic violations in foreign-accented speech. The 16th Australasian International Conference on Speech Science and Technology (SST2016). 2016.

[30] Porretta V, Tremblay A, Bolger P. Got experience? PMN amplitudes to foreign-accented speech modulated by listener experience. J Neurolinguistics. Pergamon; 2017;44: 54–67. 10.1016/J.JNEUROLING.2017.03.002

[31] Chen H, Xu Rattanasone N, Cox F, Demuth K. Effect of early dialectal exposure on adult perception of phonemic vowel length. J Acoust Soc Am. 2017;142: 1707–1716. 10.1121/1.4995994 28964051 10.1121/1.4995994

[32] Oldfield RC. The assessment and analysis of handedness: The Edinburgh inventory. Neuropsychologia. 1971;9: 97–113. 10.1016/0028-3932(71)90067-4 5146491

[33] Xu N, Demuth K. Acoustic analysis of English codas by Mandarin learners of English. In: Cox F, Demuth K, Lin S, Miles K, Palethorpe S, Shaw J, et al., editors. Proceedings of the 14th Australasian International Conference on Speech Science and Technology. Sydney, Australia: The Australasian Speech Science and Technology Association Incorporated; 2012. pp. 41–44. Available: http://assta.org/sst/SST-12/SST2012/PDF/AUTHOR/ST120082.PDF

[34] van Heuven WJB, Mandera P, Keuleers E, Brysbaert M. SUBTLEX-UK: A new and improved word frequency database for British English. Q J Exp Psychol. 2014;67: 1176–1190. 10.1080/17470218.2013.850521 24417251 10.1080/17470218.2013.850521

[35] Mok P, Dellwo V. Comparing native and non-native speech rhythm using acoustic rhythmic measures: Cantonese, Beijing Mandarin and English. In Proceedings of Speech Prosody 2008 5 6 (Vol. 4, pp. 423–426).

[36] Boersma P, Weenink D. Praat: doing phonetics by computer [Internet]. 2015. Available: http://www.praat.org/

[37] Hasting AS, Kotz SA. Speeding up syntax: On the relative timing and automaticity of local phrase structure and morphosyntactic processing as reflected in event-related brain potentials. J Cogn Neurosci. 2008;20: 1207–1219. 10.1162/jocn.2008.20083 18284341 10.1162/jocn.2008.20083

[38] Royle P, Drury JE, Steinhauer K. ERPs and task effects in the auditory processing of gender agreement and semantics in French. Ment Lex. 2013;8: 216–244. 10.1075/ml.8.2.05roy

[39] Wechsler D, Coalson DL, Raiford SE. Wechsler adult intelligence scale–Fourth Edition (WAIS–IV). San Antonio. 2008;TX: NCS Pearson. Available: http://scholar.google.com/scholar?hl=en&btnG=Search&q=intitle:Wechsler+Adult+Intelligence+Scale+-+3rd+edition#3%0Ahttp://www.statisticssolutions.com/academic-solutions/resources/directory-of-survey-instruments/wechsler-adult-intelligence-scale-fourth-edit

[40] Wakabayashi A, Baron-Cohen S, Wheelwright S, Goldenfeld N, Delaney J, Fine D, et al Development of short forms of the Empathy Quotient (EQ-Short) and the Systemizing Quotient (SQ-Short). Pers Individ Dif. 2006;41: 929–940. 10.1016/j.paid.2006.03.017

[41] Chatrian GE, Lettich E, Nelson PL. Ten Percent Electrode System for Topographic Studies of Spontaneous and Evoked EEG Activities. Am J EEG Technol. Taylor & Francis; 1985;25: 83–92. 10.1080/00029238.1985.11080163

[42] Oostenveld R, Fries P, Maris E, Schoffelen J-M. FieldTrip: Open Source Software for Advanced Analysis of MEG, EEG, and Invasive Electrophysiological Data. Comput Intell Neurosci. 2011;2011: 1–9. 10.1155/2011/720971 21253357 10.1155/2011/156869 PMC3021840

[43] Jung T-P, Makeig S, Humphries C, Lee T-W, McKeown MJ, Iragui V, et al Removing electroencephalographic artifacts by blind source separation Psychophysiology. Blackwell Publishing; 2000;37: 163–178. 10.1111/1469-8986.3720163 10731767

[44] Maris E, Oostenveld R. Nonparametric statistical testing of EEG- and MEG-data. J Neurosci Methods. 2007;164: 177–190. 10.1016/j.jneumeth.2007.03.024 17517438 10.1016/j.jneumeth.2007.03.024

[45] Luck SJ, Gaspelin N. How to get statistically significant effects in any ERP experiment (and why you shouldn’t). Psychophysiology. 2017;54: 146–157. 10.1111/psyp.12639 28000253 10.1111/psyp.12639 PMC5178877

[46] Bates D, Mächler M, Bolker B, Walker S. Fitting linear mixed-effects models using lme4. J Stat Softw. 2015;67: 51 doi: 10.18637/jss.v067.i01

[47] Kuznetsova A, Brockhoff PB, Christensen RHB. lmerTest Package: Tests in Linear Mixed Effects Models. J Stat Softw. 2017;82: 1–26. doi: 10.18637/jss.v082.i13

[48] Lenth R V. Least-Squares Means: The R Package lsmeans. J Stat Softw. 2016;69: 1–33. doi: 10.18637/jss.v069.i01

[49] Ferreira F, Patson ND. The 'good enough' approach to language comprehension. Language and Linguistics Compass. 2007 3;1(1–2):71–83.

[50] Nakano H, Saron C, Swaab TY. Speech and Span: Working Memory Capacity Impacts the Use of Animacy but Not of World Knowledge during Spoken Sentence Comprehension. J Cogn Neurosci. 2010;22: 2886–2898. 10.1162/jocn.2009.21400 19929760 10.1162/jocn.2009.21400

[51] Unsworth N, Heitz RP, Schrock JC, Engle RW. An automated version of the operation span task. Behav Res Methods. 2005;37(3):498–505. 16405146 10.3758/bf03192720

[52] Gathercole SE, Pickering SJ, Ambridge B, Wearing H. The structure of working memory from 4 to 15 years of age. Dev Psychol. 2004;40(2):177 10.1037/0012-1649.40.2.177 14979759 10.1037/0012-1649.40.2.177

[53] Federmeier KD, McLennan DB, Ochoa E, Kutas M. The impact of semantic memory organization and sentence context information on spoken language processing by younger and older adults: An ERP study. Psychophysiology. 2002;39: 133–146. 10.1017/S0048577202001373 12212662 10.1017/S0048577202001373

[54] Dave S, Brothers TA, Traxler MJ, Ferreira F, Henderson JM, Swaab TY. Electrophysiological evidence for preserved primacy of lexical prediction in aging. Neuropsychologia. 2018 8 1;117:135–47. 10.1016/j.neuropsychologia.2018.05.023 29852201 10.1016/j.neuropsychologia.2018.05.023

[55] Payne BR, Federmeier KD. Contextual constraints on lexico-semantic processing in aging: Evidence from single-word event-related brain potentials. Brain research. 2018 5 15;1687:117–28. 10.1016/j.brainres.2018.02.021 29462609 10.1016/j.brainres.2018.02.021 PMC5918631

