# Supplementary material for: Listener characteristics modulate the semantic processing of native vs. foreign-accented speech
Source: PLoS One. 2018 Dec 5;13(12):e0207452. doi: 10.1371/journal.pone.0207452 (PMC6281179; doi:10.1371/journal.pone.0207452)
Supplement: S1 Table — (DOCX) [file pone.0207452.s001.docx]

| *Semantically* *congruent* | *Semantic violation* |
| --- | --- |
| The lady bites the cupcake. | The lady bites the suitcase. |
| The barman cools the cocktail. | The barman cools the carpet. |
| The helper cuts the carrot. | The helper cuts the buses. |
| The mother feeds the baby. | The mother feeds the doorbell. |
| The butler fills the teapot. | The butler fills the carrot. |
| The builders hang the picture. | The builders hang the salad. |
| The doctor heals the soldier. | The doctor heals the cottage. |
| The fighters hit the target. | The fighters hit the daylight. |
| The captain leads the soldiers. | The captain leads the teacup. |
| The actor leaves the party. | The actor leaves the ceiling. |
| The farmers light the torches. | The farmers light the cabbage. |
| The owners lock the cottage. | The owners lock the dinner. |
| The teachers mark the pages. | The teachers mark the summer. |
| The sailor meets the captain. | The sailor meets the painting. |
| The father moves the table. | The father moves the sunshine. |
| The nanny packs the suitcase. | The nanny packs the colour. |
| The teachers park the buses. | The teachers park the pages. |
| The uncle rides the camel. | The uncle rides the season. |
| The owner rolls the carpet. | The owner rolls the birthday. |
| The sisters save the kitten. | The sisters save the puddle. |
| The doctor signs the papers. | The doctor signs the curry. |
| The nurses soak the bandage. | The nurses soak the cocktail. |
| The sisters toss the salad. | The sisters toss the siren. |
| The daughters wash the dishes. | The daughters wash the danger. |
| The women wipe the table. | The women wipe the concert. |
