# Supplementary figures and images for: Listener characteristics modulate the semantic processing of native vs. foreign-accented speech
Source: PLoS One. 2018 Dec 5;13(12):e0207452. doi: 10.1371/journal.pone.0207452 (PMC6281179; doi:10.1371/journal.pone.0207452)

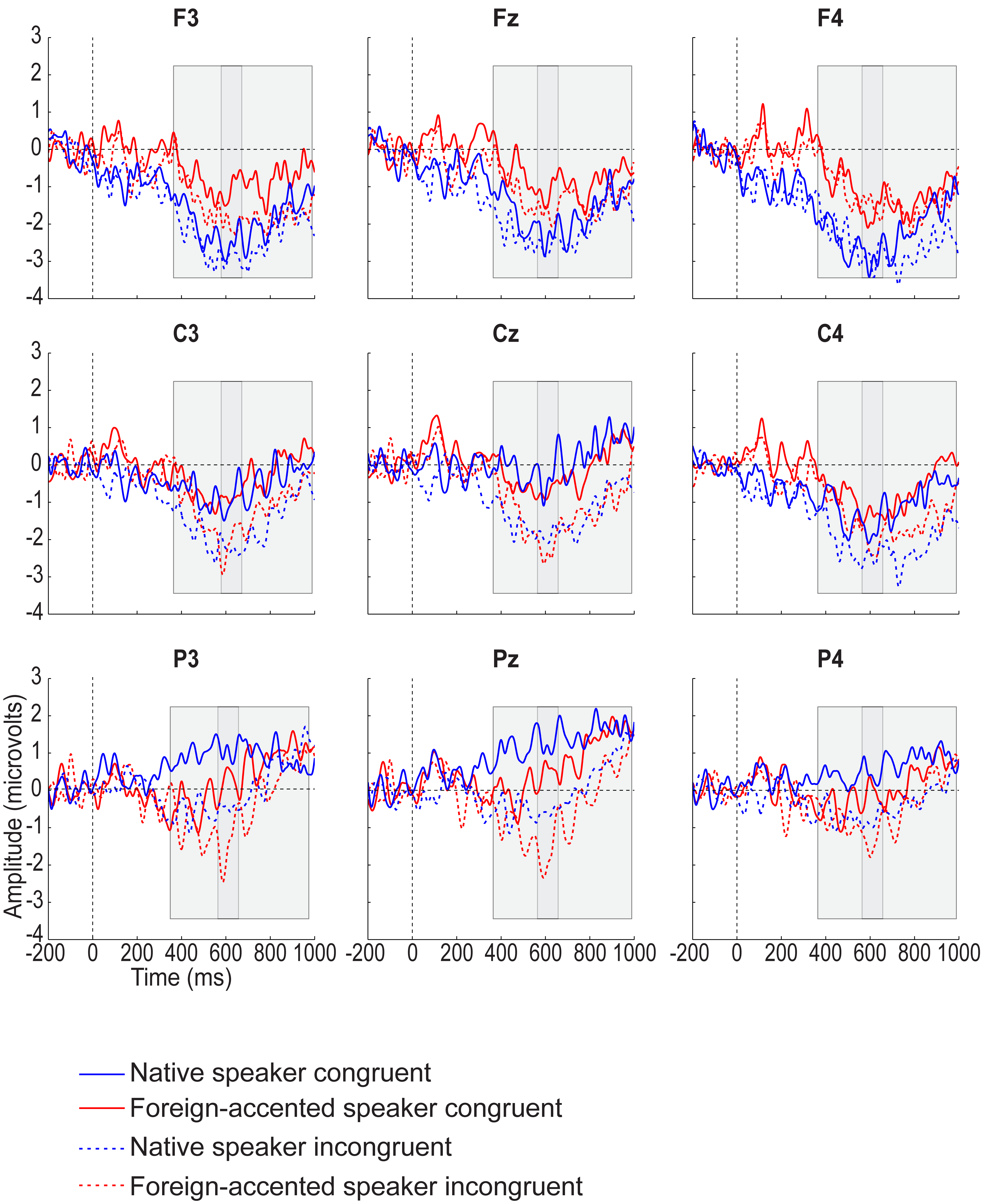

Supplement: S1 Fig — Negativity is plotted downwards. Grey boxes represent the time periods of the significant differences between congruent and incongruent sentences as shown by cluster-based permutation tests. (TIF) [file pone.0207452.s002.tif]

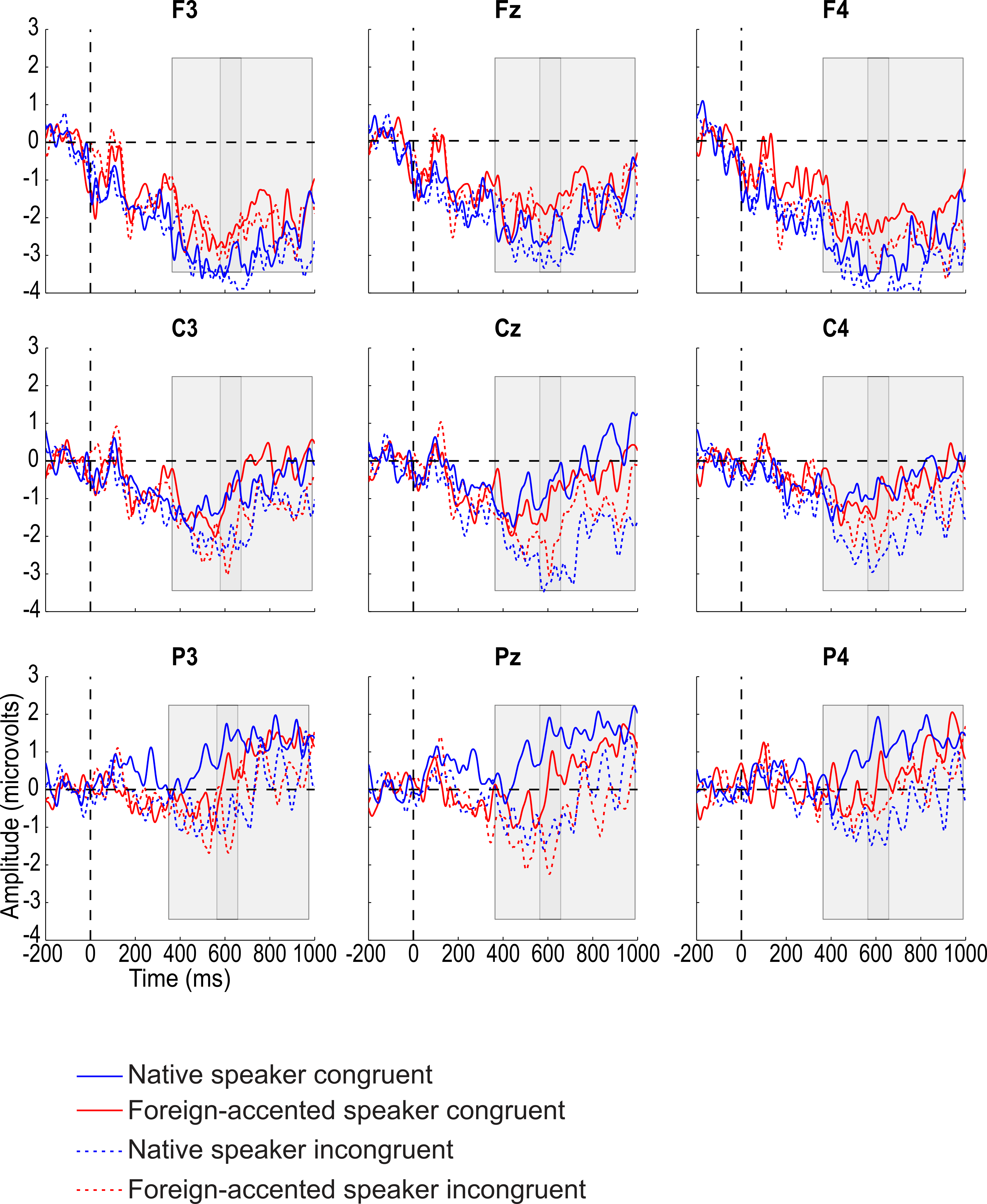

Supplement: S2 Fig — Negativity is plotted downwards. Grey boxes represent the time periods of the significant differences between congruent and incongruent sentences as shown by cluster-based permutation tests. (TIF) [file pone.0207452.s003.tif]
